# Malingering in Psychiatric Admission. Safety vs. Secondary Gain

**DOI:** 10.1192/j.eurpsy.2026.11329

**Published:** 2026-07-31

**Authors:** A. Chunarova, V. Migliaccio, H. Raai

**Affiliations:** Psychiatry, SBH Health System, NYC, United States

## Abstract

**Introduction:**

Due to an increase in patients being well-versed in healthcare system processes, there is a growing risk of individuals using the system for secondary gain. A common way patients malinger is through conditional suicidality. This places a burden on hospital resources and presents a unique clinical and legal dilemma for psychiatrists, particularly when rapid assessment and decision-making are required in the emergency department (ED). It also increases the risk of exposing patients to medications—such as antipsychotics—that carry significant cardiovascular and metabolic side effects. Management of these patients is not always straightforward, even when there is a high suspicion of malingering. The situation becomes more complex when a patient has a significant past medical and psychiatric history. The absence of precise diagnostic tools in psychiatry leaves such decisions largely to the clinical judgment and experience of the psychiatrist. (1) 1. Zwick T, Sharp C, Severn D, et al. Malingering in the emergency setting. *Cureus.* 2021;13(6):e15670. doi:10.7759/cureus.15670.

**Objectives:**

To review the current literature on assessing and managing malingering in psychiatric emergency and inpatient settings.

**Methods:**

Case presentation and literature review.

**Results:**

There are currently no standardized guidelines for assessing malingering in psychiatric settings. Most decisions are made on a case-by-case basis, relying heavily on clinical judgment, and available resources. In forensic settings, several tools are used to identify malingering or dissembling behaviors. Common assessments include the MMPI-2, PAI, M-FAST, SIRS, and SIMS. However, none are definitive on their own and should be used in conjunction with clinical evaluation. (2) (3) 2. Cassano A, Grattagliano I. Lying in the medicolegal field: malingering and psychodiagnostic assessment. *La Clinica Terapeutica.* 2019;170(2):e134-e141. doi:10.7417/CT.2019.2123.

3. Alozai UU, McPherson PK. Malingering. In: StatPearls. StatPearls Publishing; 2023.

**Conclusions:**

Decisions regarding whether to admit a patient or to proceed with a therapeutic and administrative discharge should be made through a multidisciplinary process that includes shared decision-making with the patient. Comprehensive assessments—especially when malingering is suspected—can be time-consuming and resource-intensive. The authors recommend the development of standardized guidelines for managing malingering in psychiatric settings. These should include the use of psychometric testing and a thorough evaluation to rule out medical causes of psychiatric symptoms. Treatment often requires a holistic approach, incorporating social support (e.g., housing) and psychological therapies. (4) 4. Bass C, Halligan P. Factitious disorders and malingering: challenges for clinical assessment and management. *Lancet.* 2014;383(9926):1422-1432. doi:10.1016/S0140-6736(13)62186-8.

**Disclosure of Interest:**

None Declared

